# Effects of Nanosilver Exposure on Cholinesterase Activities, CD41, and CDF/LIF-Like Expression in ZebraFish (*Danio rerio*) Larvae

**DOI:** 10.1155/2013/205183

**Published:** 2013-08-06

**Authors:** Marzhan Myrzakhanova, Chiara Gambardella, Carla Falugi, Antonietta M. Gatti, Grazia Tagliafierro, Paola Ramoino, Paolo Bianchini, Alberto Diaspro

**Affiliations:** ^1^Kokshetau State University Named after Sh. Ualikhanov, Abai Street 76, Kokshetau 020000, Kazakhstan; ^2^DISTAV, Università di Genova, Viale Benedetto XV, 16132 Genova, Italy; ^3^Nanodiagnostics Srl, Via Enrico Fermi 1/L, 41057 San Vito di Spilamberto, Italy; ^4^Nanophysics, Istituto Italiano di Tecnologia, Via Morego 30, 16163 Genova, Italy

## Abstract

Metal nanosolicoparticles are suspected to cause diseases in a number of organisms, including man. In this paper, we report the effects of nanosilver (Ag, 1–20 nm particles) on the early development of the zebrafish, a well-established vertebrate model. Embryos at the midgastrula stage were exposed to concentrations ranging from 100 to 0.001 mg/L to verify the effects on different endpoints: lethality, morphology, expression of cholinergic molecules, and development of the immune system. (1) Relative risk of mortality was exponential in the range between 0.001 and 10 mg/L. Exposure to 100 mg/L caused 100% death of embryos before reaching the tail-bud stage. (2) Developmental anomalies were present in the 72 h larvae obtained from embryos exposed to nanosilver: whole body length, decreased eye dimension, and slow response to solicitation by gentle touch with a needle tip, with a significant threshold at 0.1 mg/L. (3) Dose-dependent inhibition of acetylcholinesterase activity was significant among the exposures, except between 1 mg/L and 10 mg/L. (4) The distribution of CD41+ cells and of CDF/LIF-like immunoreactivity was altered according to the Ag concentration. The possible effect of nanosilver in impairing immune system differentiation through the inhibition of molecules related to the cholinergic system is discussed.

## 1. Introduction

One of the important problems of theoretical and practical medicine and physiology is the study of the responses of the organisms to environmental cues, with the goal of enhancing prevention of the main diseases induced by environmental stress.

An emerging risk is represented by the wide diffusion of nanoparticles, such as the silver nanoparticles, which were among the first metal nanoparticles to reach the market. Manufacturers have exploited their exceptionally efficient antibacterial, antiviral, and antifungal activity [1, 2]. As reported in the reviews [3, 4], nowadays the products containing nanosilver are increasing, as well as their worldwide diffusion for industrial processes and treatments. In daily life, consumers may have nanosilver containing room sprays, laundry detergents, toys, clothing, washing machines coatings, water purificants, and wall paint. Their high catalytic activity is due to the particularly small size (from 1 to 20 nm), that highly increases the metal surfaces [1]. Nevertheless, exposure to silver nanoparticles has been associated with “inflammatory, oxidative, genotoxic, and cytotoxic consequences” [5]. According to these authors, the particles primarily accumulate in the liver and have also been shown [6] to be toxic in other organs including the brain. Thus, the balance between the advantages and risks of nanosilver employment as a water disinfecting agent is puzzling, in part due to the scarcity of validated alternative models for testing its behavior in the aquatic environment and the effects of chronic exposures.

The first thoroughly experimented studies regard the health effects of airborne nanoparticles [8], while very few is known about the behaviour of nanoparticles in the aquatic medium, so that no databases are presently available about their presence in the aquatic environment.

In this work, we have used the first developmental stages of the cyprinid zebrafish, *Brachydanio rerio* (*Danio rerio*), as a vertebrate model to test the effects of nanosilver in the aquatic environment. In this work, we will use the abbreviation ZF for zebrafish. During the last decades, this fish has been chosen as a reliable vertebrate model for understanding basic events in developmental biology.

This model possesses a high degree of homology to the human genome [9], is easy to breed in amateur aquaria, and has transparent eggs allowing the observation of embryonic development. In 48 h after fertilization, the larva is ready to hatch and development time may be easily controlled, as fertilization occurs at the first daylight.

In this model, the effects of exposures to nanosilver have been studied by several authors: silver gills toxicity was demonstrated to be effect of particulates as opposed to release of dissolved ions [9, 10]. Bar-Ilan et al. [9] demonstrated that various sized gold and silver particles were able to affect zebrafish development, causing lethality and developmental anomalies in body axis shape and heart functionality.

In aquatic organisms, pollutants are taken up by entering food, gills, and skin and rapidly are diffused to blood and distributed to all the body regions, as demonstrated in sea urchin model [11]: ingested nanoparticles were rapidly distributed to the coelomic fluids, where impaired the immune cells morphology and activity of AChE and pseudoChE. 

For authors working on human health and mammalian models, the relationships between potentiation against impairment in immune system exerted by nanosilver are still controversial [12, 13]. We tried to bring a contribution by analysing in control and nanosilver exposed ZF embryos and larvae, the distribution pattern of immunoreactivity (IR) against the cholinergic differentiation factor/leukemia inhibiting factor (CDF/LIF), a cytokine molecule responsible for cholinergic differentiation and cholinesterase expression [14], and CD41+ thymocyte recruitment [14–16] as well. Actually, the interdependence of the lymphatic system with neural regulation was previously demonstrated in high vertebrates [17–21].

In this light, we also identified CD41 immunoreactive (IR) cells, which have been reported to be responsible to generate RAG2+ T lymphocyte precursors [22] as well as the expression of cholinesterases, which are well-known markers of cholinergic differentiation. 

## 2. Materials and Methods

Adult specimens of *Danio rerio *were purchased from a commercial source and reared in aquaria with 15 L ultrafiltered (mesh = 0.22 *μ*m) freshwater. The animals were regularly fed and maintained at temperature ranging between 24 and 28°C, with natural light/dark cycle.

The eggs, spawned and fertilized at the first daylight, were laid in trays covered with glass spheres at the bottom of aquaria. The glass spheres were used to prevent predation of eggs and embryos by adult specimens. Fertilized eggs and embryos were collected and allowed to further develop for 12 h at *T* = 28°C.

### 2.1. Characterization of Ag NPs

Ag NPs were obtained from Polytech (Germany, type WM 1000-c), supplied as a 1000 ppm in deionized water suspension of metallic silver (Ag particles encapsulated in liposomes) with an NP size between 1 and 10 nm. This “nanosuspension” did not need further sonication, since it was very stable and it did not form any agglomerates [23]. Size range and zeta potential of Ag NPs were evaluated by dynamic light scattering (DLS) analysis (Malvern, UK). 

### 2.2. Exposure

At 12 h dead and anomalous embryos were discarded, and the healthy ones were divided into multiwell dishes containing nanosilver particles (Ag) suspended in ultrafiltered freshwater at concentrations ranging from 100 to 0.001 mg/L.

Control eggs were maintained in ultrafiltered fresh water for the time of the experiment. 

All the dishes were placed in a thermostat at *T* = 25°C and the control and exposed specimens were allowed to develop for further 24 and 48 h, up to hatching. 

After those times, the survived larvae were counted and measured. Developmental anomalies were registered and classified.

### 2.3. Fixation for Immunochemistry Reactions

After incubation, the living larvae were fixed in paraformaldehyde (PFA) 3% in phosphate buffer saline (PBS) + 70% cold methanol for 20 min and then rinsed and rehydrated in 0.1% BSA/PBS for 10 min before processing for immunohistochemical reactions.

### 2.4. Localization of Molecules Immunologically Related to CDF/LIF and to CD41

Samples were incubated overnight at *T* = 5°C in the primary antibodies diluted 1 : 200 in PBS containing 0.5% BSA, 0.1% NGS. The primary antibodies were anti-leukemia inhibitory factor (CDF/LIF), raised in goat (Sigma, IT), or anti-CD41 (ABCAM, IT, l1024) raised in mouse. After rinsing in PBS/BSA, the samples were incubated overnight at 5°C in the secondary antibodies (chick anti-goat Alexafluor 488 and rabbit anti-mouse igG, resp.), 1: 300 in PBS/BSA. Nuclei were counterstained with 1 *μ*M RNAase followed by 2 *μ*M propidium iodide (PI). The preparations were mounted on a slide with the antifading gelvatol [24]. Images (1024 × 1024 × 8 bit) were acquired on a Leica TCS SP5 AOBS confocal laser scanning microscope (Leica Microsystems Mannheim, Germany), using a one airy disk unit pinhole diameter and an HCX PL APO 20x/0.70 objective; magnifications were obtained by scan field selection. Alexa Fluor 488 was excited with the 488 nm line of the Ar laser, and its fluorescence was collected in a spectral window of 500–530 nm. Propidium iodide was excited with the 543 line of the He-Ne laser, and its fluorescence was collected in a spectral window of 600–640 nm. Laser scanning transmitted light images were obtained using the 488 nm laser line.

### 2.5. Quantitative Analysis of ChE Activity

The control and exposed larvae (a dry volume of 100 *μ*L for each vial) were frozen in 100 *μ*L of 0.1 M phosphate buffer, pH7 for 24 h. Then, they were brought to the room temperature and homogenate in an ice bath by use of a minipotter homogenisator (Braun-Melsungen). The protein content of each sample was determined by the Bradford method. Of these, a quantity corresponding to 30 mg protein was used for the measurement of ChE. The lecture of the speed of substrates cleavage was performed in 0.1 phosphate buffer, pH 8, in the presence of dithiobis nitrobenzoic acid (DTNB) and bicarbonate, according to the quantitative method suggested by Ellman et al. [7], and by use of a spectrophotometer JENWAY, 6405 UV/vis, at *λ* = 412 nm. The absorbance kinetics were recorded for 3 min, for at least 15 homogenate fractions/each exposure. The amount of cholinesterase units (ChE U) was related to the protein content of each sample (30 mg) previously measured by the Bradford method. (one ChE U hydrolyzes 1 *μ*mole of substrate per min at pH 8.0 at 37°C).

Acetylthiocholine iodide (ATCh), acetyl(*β*-methyl)thiocholine iodide (AMTCh), 1,5-bis(4-allyldimethylammoniumphenyl)-pentan-3-one dibromide (BW284C51), Tetraisopropyl pyro phosphoramide (iso-OMPA), propionylthiocholine iodide (PTCh), S-butyrylthiocholine iodide (BTCh), 5,5-dithiobis(2-nitrobenzoic acid) (DTNB), and choline chloride were purchased from Sigma.

### 2.6. Histochemical Localization of ChE Activity [25]

The larvae were fixed in paraformaldehyde 4% in ultrafiltered tapwater and incubated in the mixture suggested by Karnovsky and Roots [25], containing 0.03 M copper sulphate,, 0.005 M K ferricyanide, and 0.1 M natrium citrate in 0.1 M maleate buffer. AMTCh was used as a substrate for AChE reaction. The incubation was carried out at room temperature, and the development of the reaction was monitored on a light microscope. The brown-magenta staining was due to the precipitation of metallic salts at the enzymic sites.

### 2.7. Specificity Controls

CDF/LIF-like and CD41-like immunoreactivity (IR) controls were performed by omitting the primary antibody. Aspecific reactions were blocked by use of BSA, NGS, as above described.

The specificity of ChE enzyme activity was checked by AcTCh, and Ac*β*MeTCh and BuSCh were selected as diagnostic substrates selective for ChE, AChE, and BChE, respectively [26]. A further specificity diagnosis was carried out by exposing homogenates before and during incubation in the Ellman et al. [7] medium to specific inhibitors, 10^−4^ M BW284c51 (specific inhibitor of AChE), 10^−4^ M eserine (inhibitor of AChE and pseudocholinesterases), and 10^−4^ M iso-OMPA (tetra isopropyl pyrophosphoramide, specific inhibitor of BChE). All the inhibitors were obtained by Sigma (IT).

### 2.8. Statistical Analysis

#### 2.8.1. Lethality

Although the statistic elaboration of mortality data (ANOVA) did not show significant dose dependence, nevertheless a strong trend was seen for the effects exerted by the exposure to the different Ag concentrations. Thus, we calculated the relative risk (RR), showing the probability of death occurring in the exposed group versus te control cohort of larvae. RR shows the probability of death in the exposed groups versus nonexposed groups [27]. With this method, the nonexposed group (control) is always 1, because it is divided for itself, whichever is the occurrence (from 0 to 100%) of the event inside it. For example, if the occurrence of the death is 35% in the control cohort, and 45% in one exposed cohort the risk ratio for the controls will be 35/35 = 1, while it will be 45/35, that is, 1.28 times major risk of death in exposed samples. 

### 2.9. Homogeneous Measures

For homogeneous measures (body length, enzyme activity), a one-way ANOVA was performed to test for differences among the effects of different nanosilver concentrations. Prior to running analyses, homogeneity of variances was tested by Levene's test; whenever necessary, data were transformed and retested. When transformation did not produce homogeneous variances, we set *a* = 0.01, in order to make up for the increased likelihood of type 1 error [28]. Post hoc multiple comparisons after ANOVA were made by Tukey's test of honestly significant differences. Descriptive statistics are reported as mean ± standard deviation. All analyses were performed using the free PAST software package version 2.17c [29].

## 3. Results

### 3.1. Effects of Exposures on Survival of Embryos and Larvae

**Figure 1:**
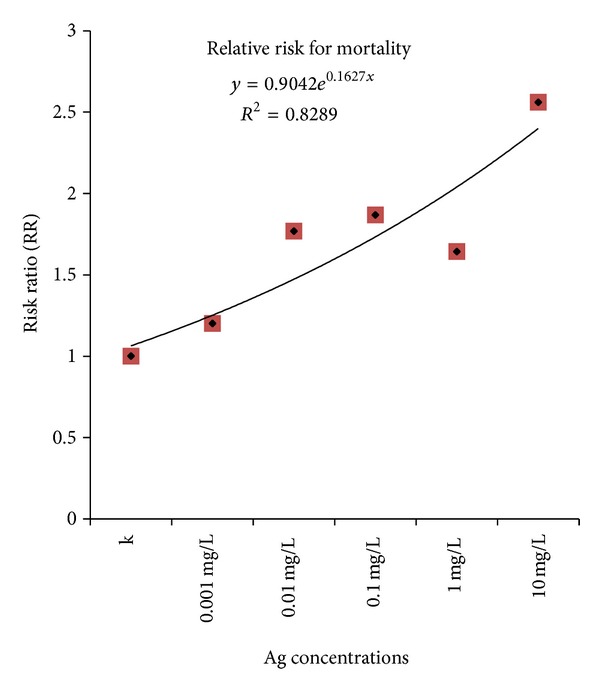
Death risk probability (RR) according to the different Ag concentrations (0, 0.001, 0.01, 0.1, 1, and 10 mg/L). *x*-axis = Ag concentrations; *y*-axis = risk ratio with respect to unexposed samples, assumed as 1.

The occurrence of dead embryos for each exposure was different among the embryos exposed to the different Ag concentrations, with a trend to increase from control to the more concentrated exposures, but no homogeneity among the different groups of embryos was present. The RR elaboration of the data showed an exponential risk probability of about 1.2-folds with respect to the control for the exposure to 0.001 mg/L, average risk of 1.7-folds for the embryos exposed to 0.01 mg/L, 0.1 mg/L, and 1 mg/L, and a risk of 2.56-folds for the exposures to 10 mg/L Ag concentration (Figure 1). The exposure to 100 mg/L caused 100% death in almost all the experiments.

### 3.2. Morphological and Behavioral Effects of the 48 h Exposures

**Figure 2:**
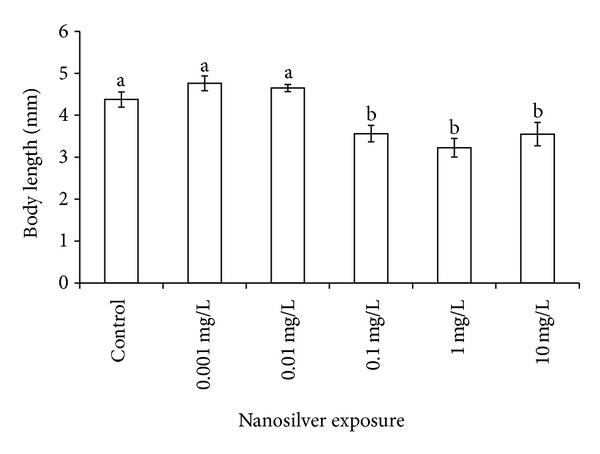
Morphological effects of Ag exposure on body length. Differences among groups are indicated by different letters above histograms. *x*-axis = Ag concentrations; *y*-axis = body length from the mouth to the tail tip.

**Figure 3:**
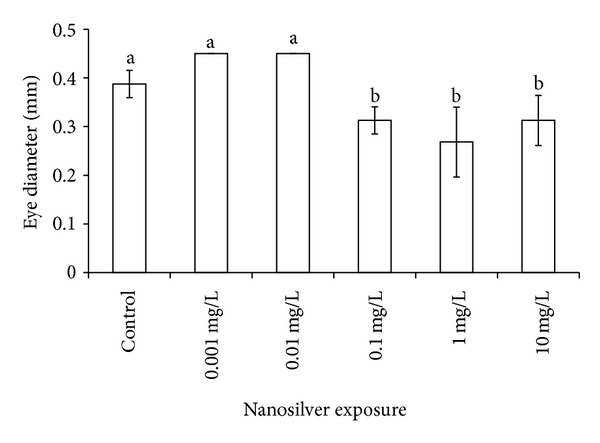
Morphological effects of Ag exposure on eye diameter. Symbols as in Figure 2.

Exposed samples presented some developmental anomalies, including scarce reactivity to stimulation exerted by the gentle touch of a needle tip. If after the third touch they did not move or their swim was inefficient, we marked a no-reply in the excel column used for registration. This feature was not dose dependent: control = 17%: 0.001 = 44%; 0.01 and 0.1 mg/L = 0%; 1 mg/L = 75% and 10 mg/L = 100%. The anomalies were classified as anomalous shape of the vertebrate column, anomalies in the otoliths shape and size (not shown). The most measurable effects were represented by the larval length (Figure 2) and by the diameter of the eyes (Figure 3). These showed a significant (*P* ≪ 0.01) threshold of effect at 0.1 mg/L, as shown in Figures 2 and 3.

### 3.3. Cholinesterase Activity Quantitative Assay [7]

**Figure 4:**
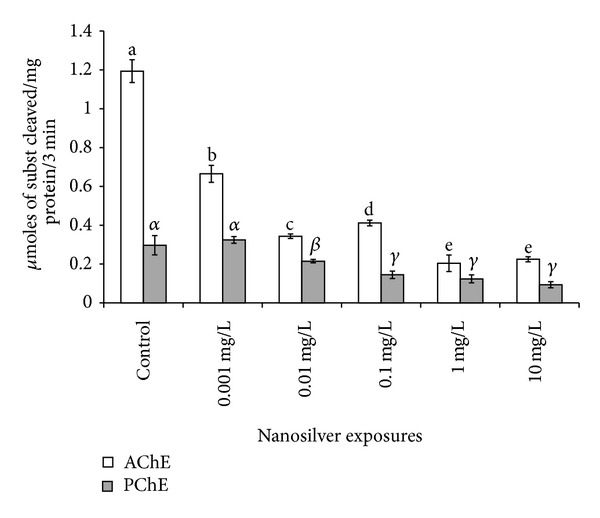
Effect of Ag exposures on cholinesterase activities. Symbols as in Figure 2. The symbols over the diagram are different for significant differences: for AChE (Latin letters) the dose dependence is significant among all the the concentrations, except the last two, while a significant threshold of effect is present for PChE (Greek letters) between 0.001 and 0.01 mg/L and between 0.01 and 0.1 mg/L Ag suspension. PChE shows the presence of aspecific enzymes able to cleave pTChI.

Both acetylcholinesterase and propionylcholinesterase activities were decreased by exposures; PChE activity was always lower than that AChE (Figure 4). The AChE activity (measured by the cleavage speed of AcTChI) was always significantly inhibited by the exposure to Ag, at any concentration. In addition, a growing enzyme inhibition was present among the different exposures (*P* < 0.01), in a dose-dependent way, except between the exposures to 1 and 10 mg/L Ag. 

Pseudocholinesterase (PChE) activity, measured by cleavage of PTChI, showed nondose-dependent decrease caused by exposure to crescent Ag concentrations, as 0.001 mg/L and controls did not differ in a significant way, while the exposure to 0.01 and 0.1 mg/L caused significantly different impairment (*P* < 0.01), and no significant difference was present among 0.1, 1, and 10 mg/L exposures (Figure 4). 

### 3.4. Specificity Control of the Enzyme Activities (Figure 5)

Acetylcholinesterase activity measured by cleavage of AcTChI was impaired by BW284c51 (activity reduced by 90% in controls), while 10^−4^ M iso-OMPA affected the speed by a negligible percentage (activity reduced by 9.6% in controls). The inhibition percentages exerted by BW284 c51 decreased with Ag concentration, suggesting an increased amount of BChE in parallel with the decrease of AChE activity (Figure 6).

### 3.5. Localization of Molecules Immunologically Related to CDF/LIF (Figure 7)

In control samples, the CDF/LIF-like fluorescent immune reaction marked a complex net of vessels in the head, in the thymus (not shown), and the main trunk vessels. 

For simplicity, we showed only the trunk and tail regions, containing the supraintestinal (SILV), the dorsal longitudinal (DLLV), the paracordal (PCLV), and the intersegmental lymphatic vessel (ISLV) (according to the anatomical description by Meeker and Trede [30]). In control larvae, CDF/LIF IR defined the particular structure of the vessels and of the varicosities, and the thin intersegmental vessels (ISLV), joining the DLLV. CDF/LIF-like IR also stained the walls of the vessels and molecules released among the muscle fibres (Figure 7(a)). The samples exposed to 0.001 mg/L nanosilver showed an aspect very similar to the controls; IR-positive cells were present inside the PCLV varicosities and ISLVs departing from them (Figure 7(b)). The samples exposed to higher concentrations of Ag showed decreasing distribution of positive sites (Figures 7(c), 7(d), and 7(e)). The ISLVs were not decored in the samples exposed to 0.01 and 0.1 mg/L (Figures 7(c) and 7(d)); in the PCLV the LIF IR appeared irregularly distributed and weaker in the samples exposed to 1 mg Ag/L (Figure 7(e)). Traces of released LIF-like molecules were seen inside the ISLVs in samples exposed to 1 mg/L, while the samples exposed to 10 mg/L only showed IR traces, scattered inside the DLLV, the SILV, and the PCLV (Figure 7(f)).

### 3.6. *In Toto* Staining of AChE Activity (Karnovski and Roots [25])

Figures 8 and 9 show the localization of AChE activity, revealed by use of AcMTCh as a substrate in a ZF control larvae. 

### 3.7. Localization of Molecules Immunologically Related to CD 41 (Figure 10)

Cells showing IR to CD41 were present in the lymphatic vessels of controls and of the larvae obtained from embryos exposed to 0.001 and 0.01 mg/L (Figures 10(a), 10(b), and 10(f)), including the PCLV and the ISLV. The PCLV presented along its length varicosities (V), full of cells. The positive IR progressively disappeared from the cells contained in the ISLV starting from the exposure to 0.1 mg/L (Figures 10(c) and 10(d)) and remained localized in cell groups scattered along the PCLV and the SILV in the larvae exposed to 1 and 10 mg Ag/L (Figures 10(e) and 10(f)).

## 4. Discussion

Our results show a toxic effect and a death risk exerted by nanosilver exposure, inhibition of AChE and PseudoChE activity, and impaired recruitment of T-lymphocytes (CD41+ cells). The lethal effects showed a random occurrence, so that lethal toxicity was only demonstrated for the Ag suspension of 100 mg/L. Nevertheless, the risk of death showed a trend growing exponentially with the increasing concentration of the Ag suspension. As it concerns the morphophysiological and enzymatic responses to exposure, the controls and the samples exposed to 0.001 mg Ag/L generally presented similar effects, while the exposures to 0.01 and 0.1 mg/L caused statistically significant (*P* < 0.001) responses, as compared to controls, followed by the effects of the exposures to 1 mg/L and 10 mg/L. This suggests the presence of thresholds for the Ag effects, probably corresponding to stages of increased catalytic activity. At these concentrations, the total area of the particles surface would reach the ability to interfere in the normal chemical reactions of the organisms, such as for instance the activity of key enzymes. These thresholds were rather congruent, although slightly different among the different endpoints. The effects found in exposed larvae were mainly due to the particulate nanosilver suspension other than to the possible ionic component. Although no news are available about the Ag^(+)^ component, the encapsulation in liposomes prevents direct contact with water. Besides, pH of freshwater is neutral, and ionic transformation is proportional to the alkalinity of the medium, as reported by [31] and by A.G. Oromieh (http://stud.epsilon.slu.se/2358/1/geranmayeh_oromieh_a_110315.pdf). 

### 4.1. Effects on Cholinesterase Activities

In vertebrates, acetylcholinesterase plays a key role in the modulation of neuromuscular impulse transmission, while pseudocholinesterases are mainly represented in blood plasma [32]. The role of AChE is to remove acetylcholine (ACh), the cholinergic signal molecule, from its receptors (AChRs) in the plasma membrane of the target cells. In the zebrafish early larva, the cholinergic tissues are mostly represented by the heart and the neuromuscular junctions and are scarcely present in the head and brain (see Figure 8). The tissues are characterized by different kinds of AChRs. In the neuromuscular tissue, the nicotinic AChRs are preeminent and associated to Na^+^ selective channels, while in the other tissues the muscarinic ones are mostly represented. These last are membrane receptors, associated to G-proteins in the intracellular domain, triggering a second messengers cascade. This cascade culminates in both nuclear and cytoplasmic events, leading to differentiative (through cGMP and protein kinases) or electrical events. In particular, the electrical events are related to the IP3 release and calcium dynamics (m1 and m3 AChRs) or to K^+^ outwards fluxes (m2 and m4 AChRs). In addition, AChE has been demonstrated to be a modulator of a number of nonneuromuscular events leading embryonic development [33], is a wellknown biomarker of stress [34], is involved and responsible for the control of neural and immune system interaction [35, 36], modulates cell proliferation [37] and cell migration [38], and regulates cell-to-cell communication during differentiative [39] and inflammatory events [40]. In these cases, it is able to modulate the relatively slow cell-to-cell communication related to these events, besides the high-speed signals taking place in the well established neuromuscular synapses. In some cases, such as the function in morphogenetic movements, well elucidated by Drews [38] the action of AChE may be affected by its structure of adhesion protein, which allows linking the cell glycocalyx to substrate matrices, such as fibronectin and laminin [39].

**Figure 5:**
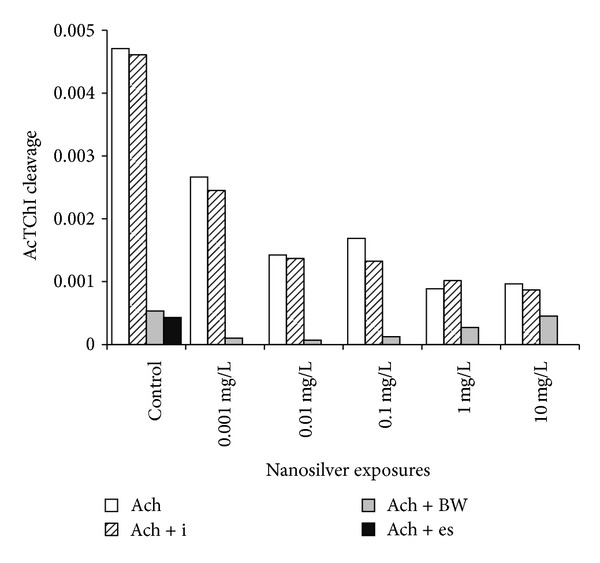
AChE impairing by selective inhibitors: Ach = AcTChI as a substrate without inhibitors; Ach + i = AcTChI + 10^−4^ M iso-OMPA, selective inhibitor of pseudocholinesterase (BChE, E.C.3.1.1.8); Ach + e = AcTChI + 10^−4^ M eserin, mainly inhibitor of AChE; Ach + BW = AcTChI + 10^−4^ M BW284c51, selective inhibitor of AChE. Eserin was used only once, for controls. No specificity control was performed on PChE activity. *y*-axis = micromoles of substrate cleaved in 3 mins.

**Figure 6:**
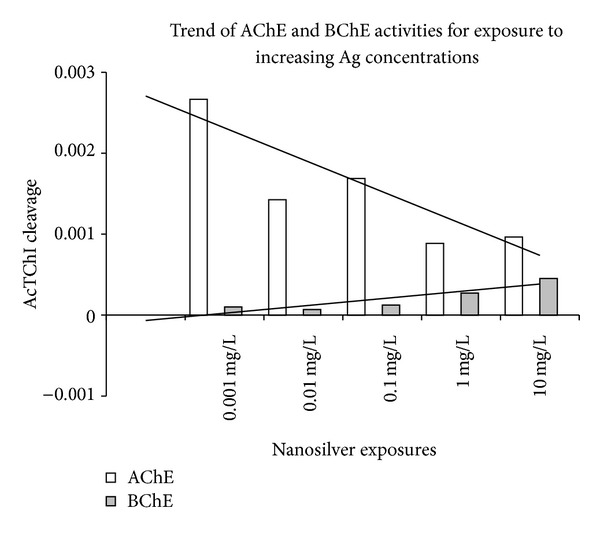
Reciprocal variation of AChE and pseudoChE activities, extrapolated from Figure 5. The term pseudocholinesterase refers to the activity remaining in samples preincubated with and incubated in the [7] medium containing 10^−5^ M BW284c51. *y*-axis = *μ*moles of AcTCh cleaved in 3 mins; white columns: no inhibitors added; grey columns: residual activity after exposure to BW284c51.

**Figure 7:**
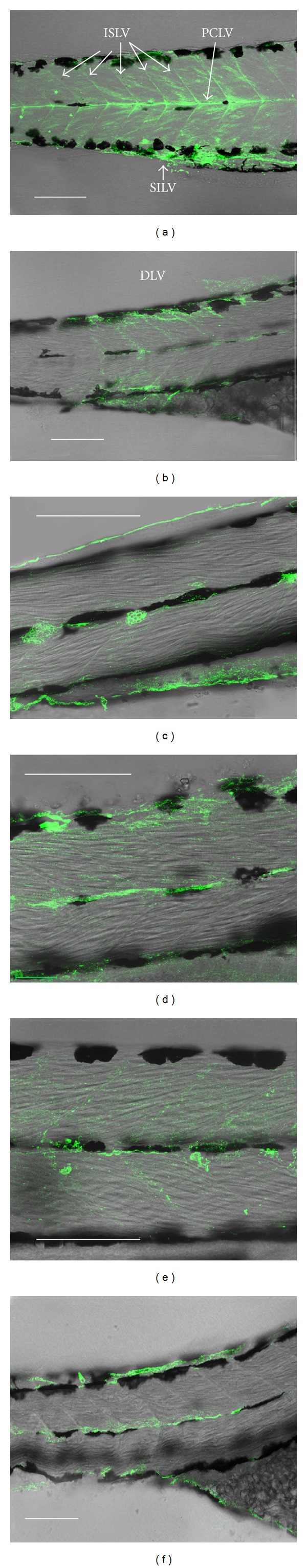
CDF/LIF IR, confocal sections of 72 h larvae, stained *in toto* by immunofluorescence. (a) Control larva; (b) Larva obtained from embryo exposed to 0.001 mg/L nanosilver; ((c), (d)) Larvae obtained from embryos exposed to 0.01 and 0.1 mg/L Ag; ((e), (f)) Larvae obtained from embryos exposed to 1 and 10 mg/L Ag, respectively.

At the studied ZF stages, ChE activities in the zebrafish seem to be mainly represented by AChE (E.C., 3.1.1.7) and, at a minor extent, by pseudocholinesterases, such as PChE, able to cleave propionylthiocholine iodide. Butyrylcholinesterase (BChE, E.C. 3.1.1.8) is scarcely present at these developmental stages as shown by the scarce inhibition of AcTChI cleavage exerted by iso-OMPA in controls (Figure 5) but seems to increase its activity while AChE activity is decreasing (Figure 6, shown by the residual activity in the presence of BW284c51). This may be due to a possible role of pseudoChEs in integrating the AChE activity failure, as it has been reported by Mesulam et al. [41] in case of AChE knockout. 

### 4.2. Possible Role of Cholinesterase in Developmental Events and Larval Health

The alteration found in the AChE and pseudoChE activities might be involved in the morphological and physiological responses of the zebrafish larvae. Actually, during the last 40 years, awareness is increased of the AChE's role in regulating cell-to-cell communications such as the ones occurring during embryonic development [38, 39], for example, inductive processes, morphogenetic cell migrations, regulation of axon growth, and neurogenesis. Moreover, ChE activities are responsible for other relevant biological events, including inflammation [40] and regulation of the balance between cell proliferation and cell death [37], as well as the modulation of cell adhesion and cell migration [38]. In particular, cholinesterase activity alteration has been shown to be impaired by NPs in sea urchin immune cells, where a reduced expression of HSP70 and GRP78 was also found [11]. This may explain the presently found effects on ZF larval morphology and on recruitment of the CD41-like IR cells. Actually, the affected larval defence seems to be decreased by the exposures. 

### 4.3. Comparison between CDF/LIF and AChE Distribution in ZF Larvae

**Figure 8:**
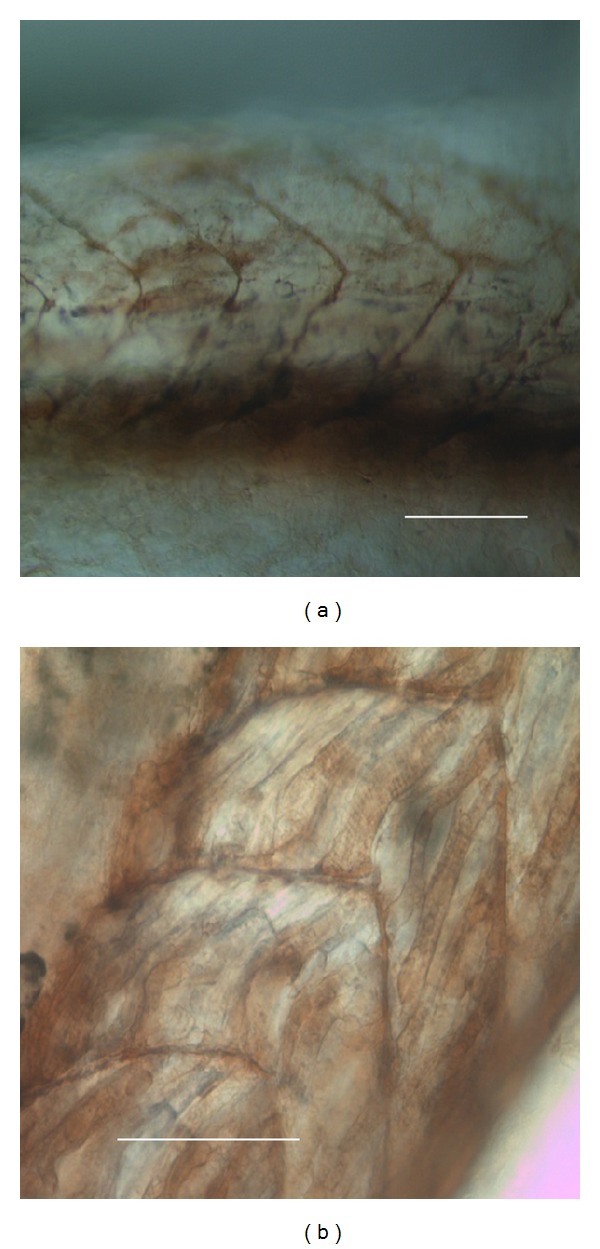
AChE activity localization in the trunk of a 48 h ZF larva. The brown-magenta precipitation shows the activity sites inside the trunk ISLV and in the striate metameric muscles.

**Figure 9:**
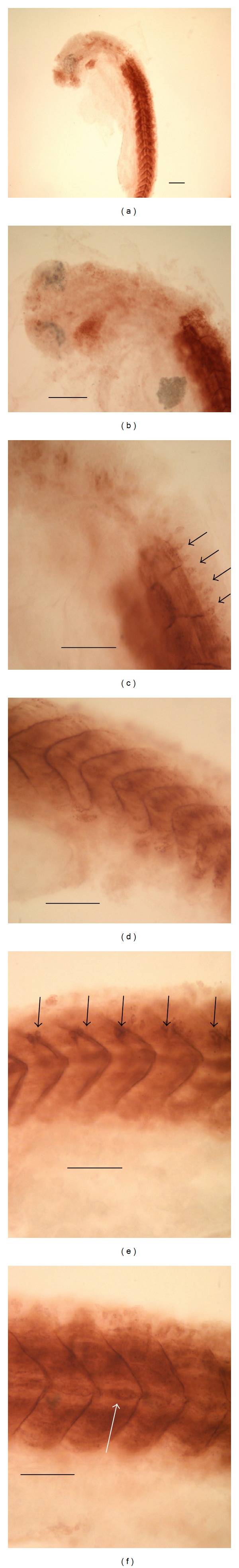
(a) 48 h old ZF larva. In the head, only olfactive areas and heart were marked, while most of the staining was concentrated in the neuromuscular sites of trunk and tail. (b) A higher magnification of the head; (c) The arrows point to motor single neurons in the ventral part of the neural tube; (d) a particular of the tail, showing the staining of the myocommas; (e) the motor neurons are viewed in a slightly rotated trunk of a 72 h larva; (f) the white arrow points to the paracordal vessel of the 72 h larva.

**Figure 10:**
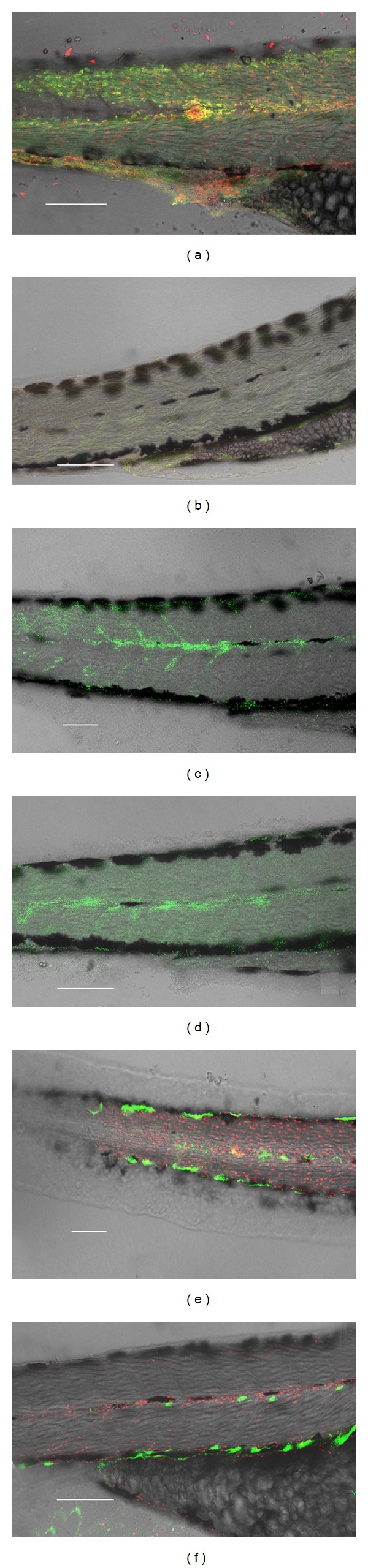
CD41 + IR, confocal sections of 72 h larvae. The green fluorescence identifies the CD41(+) cells and molecules. The red fluorescence is due to propidium iodide. (a) Unexposed sample (control); (b) Control of IR specificity (primary antibody omitted); ((c), (d)) Larvae obtained from embryos exposed to 0.01 and 0.1 mg Ag/L; ((e), (f)) Larvae obtained from embryos exposed to 1 and 10 mg Ag/L, respectively. Bar equals 100 *μ*m, the figures have the same magnification.

The distribution of AChE activity, as compared to the CDF/LIF pattern, is not completely overlapping, because in the head AChE activity is not present, except in the main sense organs (Figures 9(a) and 9(b)). In the trunk it is widely present, localized in the lymphatic vessels (Figures 8(a) and 8(b)), including the ISLV and PCLVs, where it presents the same distribution of CDF/LIF (Figures 8 and 9(f), white arrow) and in sites where it exerts its neuromuscular function, associated to the muscle, miocommas, and ventral neurons of the neural tube (Figures 9(c), 9(d), and 9(e), arrows).

### 4.4. Possible Mechanisms for the Found Effects

The small size of the NPs, ranging between 1 and 10 nm, may be responsible for enhanced penetration in cells and tissues, reaching the hematopoietic tissues, directly interfering in the cell-to cell communication modulated by cholinesterase activity, and impinging on differentiation of immune cells during the first developmental events of the zebrafish. The zebrafish CD41(+) cells are known to be involved in the immune system differentiation, because, after the first wave of aorta-gonad-mesonephros emopoyesis, these cells colonize the thymus to generate rag2(+) T-lymphocyte precursors [22]. These effects may be exerted independently from each other, but the CD41(+) cells recruitment may also depend on the modulatory action of the cholinergic system. In mice, T-lymphocyte differentiation was shown to be regulated by a cholinergic pathway [42].

The relationship between AChE activity and blood [43] and in thymocytes [44] is known since more than 20 years, and recently it was associated to stress events in several aquatic organisms, such as prawn exposed to ChE-inhibiting pesticides [45]. In general, the cholinergic anti-inflammatory system and *α*7 nicotinic receptors in macrophages have been proposed to play a role in neuroimmunomodulation of resistance and relief in mammalian inflammatory processes [46]. In the high vertebrates interrelationship between the lymphatic system cell mobilization and neurotransmitter systems was also demonstrated [16–19]. 

The decrease of AChE and PChE activity at doses from 01, 1, and 10 mg/L corresponding to the doses inhibiting the presence of CD41 IR cells suggests a possible function of these enzymes also in the regulation of thymocytes recruitment during early zebrafish development, or as suggested by Koelle et al. [47], pseudoChE could substitute the activity of AChE, when it is inhibited. 

## 5. Conclusions

The effects of exposure to NPs show a trend to impairment of immune responses, possibly related to the degree of inhibition of AChE activity. This hypothesis paves the way to further studies on the presence of molecules related to the cholinergic system in the immune cells of different organisms and the competitive effects possibly exerted by the Ag NPs. 

The general mechanism of action seems to be represented by a depression of the immune system, as previously stated by Falugi and Aluigi [39] in sea urchin immune cells, where not only ChE activity but also HSP70 and GRP78 expression was depressed. The general mechanism of nanoparticles on organismic health is represented by the rising of ROS and inflammation. This in turn causes or is followed by increased expression of acetylcholine [48]. This should be mitigated by AChE activity, whose task is to remove AChE from the receptors, but AChE activity is depressed by some concentrations of the Ag NPs. As a consequence, AChE inhibition is at the same time responsible for increasing inflammation by ACh and for the block of apoptosis, as it was demonstrated by apoptosis suppressions in models treated by AChE antisense [4], or by AChE inhibitors, such as organophosphorus compounds [49]. Other authors found that ACh itself, at high concentration, may exert ACh receptors blockade; this paradox was resolved by Dionne and Stevens [50] at the level of frog neuromuscular junctions.

As reported by different authors, the mechanism of action of the nanoparticles is largely unknown: in particular, it is not clear which are the NP characteristics triggering the organismic responses [51]. In general, nanoparticles do not seem to act by poisoning, but by their mechanical effect, that raise inflammation. The responses of the molecules related to the cholinergic system to inflammation have been studied thoroughly by other authors: the group of Wessler et al. (e.g., [48]), the group of Soreq (e.g. [52]), and others (e.g., [53]). On the other hand, CDF/LIF, which enhances the cholinergic differentiation, is involved in the downregulation of immune cell proliferation in case of inflammation. Thus the cholinergic system seems to be involved in the modulation of immune cells responses to inflammation, and AChE is the factor modulating the balance between inhibition and enhancement of the responses, as already stated for the effect of organophosphate pesticides on the sea urchin development [11]. However, the effects of exposure to nanoparticles seem to be more puzzling as compared to the exposures to cholinergic toxicants, such as organophosphates (OPs) and carbamates (CBs), although in a certain measure they appear similar. Actually, some induced anomalies (smaller eyes, bend trunk, etc., as described by [54]) are very similar. Nevertheless, the occurrence of the anomalies is not dose dependent as in the case of exposure to organophosphorus compounds, such as chlorpirifos or diazinon. This may be due to the different mode of action of the nanoparticles as compared to chemicals. In fact, the nanoparticles effect is due to their physical features (shape, size, degree of suspension, etc.), while the effect of chemicals such as OPs or their oxonized derivatives is due to irreversible chemical link between the phosphate group and the serine molecule in the catalytic site of the AChE molecule [51, 55–57]. Also the inhibition of the cholinergic system exerted by the Ag nanoparticles could be due to nonspecific steric competition at the level of the gorge, due to the mechanical accumulation that in some cases might act as a plug.. This could explain why the effect is rather uncertain, because the presence of the nanoparticles on the gorge in this case would be “casual” and present a threshold at certain concentrations. Otherwise, the change in cholinesterase activity might be due to a specific response to stress, as Ag nanoparticles were also found to induce oxidative stress in dose- and time-dependent manners indicated by depletion of GSH and induction of ROS, LPO, SOD, and catalase [58]. As thoroughly described by the group of Soreq, the AChE gene encoding the acetylcholine-hydrolyzing enzyme acetylcholinesterase is known to undergo long-lasting transcriptional and alternative splicing changes after stress [59]. In particular, a relationship between stress and hematopoiesis/inflammation events was established to interact with AChE molecular forms [43, 56]. These authors describe how stress can modify alternative splicing giving rise to AChE variants and suppressing micro-RNAs. The findings of these authors pave the way to understanding responses of the organisms to environmental cues, with the goal of enhancing prevention of the main diseases induced by environmental stress, including the scientific control of the possible medical application [60].
